# Enhancing heart failure treatment decisions: interpretable machine learning models for advanced therapy eligibility prediction using EHR data

**DOI:** 10.1186/s12911-024-02453-y

**Published:** 2024-02-14

**Authors:** Yufeng Zhang, Jessica R. Golbus, Emily Wittrup, Keith D. Aaronson, Kayvan Najarian

**Affiliations:** 1 Department of Computational Medicine and Bioinformatics, University of Michigan, Ann Arbor, 48103 MI USA; 2 Division of Cardiovascular Medicine, Department of Internal Medicine, University of Michigan, Ann Arbor, MI USA; 3 Department of Emergency Medicine, University of Michigan, Ann Arbor, MI USA; 4 Department of Electrical Engineering and Computer Science, University of Michigan, Ann Arbor, MI USA; 5 Michigan Institute for Data Science, University of Michigan, Ann Arbor, MI USA

**Keywords:** Heart failure, Electronic health records, Machine learning, Interpretability

## Abstract

Timely and accurate referral of end-stage heart failure patients for advanced therapies, including heart transplants and mechanical circulatory support, plays an important role in improving patient outcomes and saving costs. However, the decision-making process is complex, nuanced, and time-consuming, requiring cardiologists with specialized expertise and training in heart failure and transplantation.

In this study, we propose two logistic tensor regression-based models to predict patients with heart failure warranting evaluation for advanced heart failure therapies using irregularly spaced sequential electronic health records at the population and individual levels. The clinical features were collected at the previous visit and the predictions were made at the very beginning of the subsequent visit. Patient-wise ten-fold cross-validation experiments were performed. Standard LTR achieved an average F1 score of 0.708, AUC of 0.903, and AUPRC of 0.836. Personalized LTR obtained an F1 score of 0.670, an AUC of 0.869 and an AUPRC of 0.839. The two models not only outperformed all other machine learning models to which they were compared but also improved the performance and robustness of the other models via weight transfer. The AUPRC scores of support vector machine, random forest, and Naive Bayes are improved by 8.87%, 7.24%, and 11.38%, respectively.

The two models can evaluate the importance of clinical features associated with advanced therapy referral. The five most important medical codes, including chronic kidney disease, hypotension, pulmonary heart disease, mitral regurgitation, and atherosclerotic heart disease, were reviewed and validated with literature and by heart failure cardiologists. Our proposed models effectively utilize EHRs for potential advanced therapies necessity in heart failure patients while explaining the importance of comorbidities and other clinical events. The information learned from trained model training could offer further insight into risk factors contributing to the progression of heart failure at both the population and individual levels.

## Introduction

Heart failure (HF), a disease with a prevalence as high as 2% in developed countries, affects an increasing number of people each year and is projected to impact 8 million worldwide by 2030 [1, 2]. Patients with end-stage heart failure are characterized by significant structural change in the heart and prominent symptoms of heart failure [3]. Statistics show that the 1-year mortality rate for this population could be as high as 50% [4], making it a significant public health issue.

Due to the dismal prognosis of end-stage heart failure, several surgical approaches have been developed and demonstrated to improve quality of life and survival compared with traditional medical treatments. There are two major advanced therapies: heart transplantation (HT) and mechanical circulatory support (MCS). However, both approaches have potential risks and limitations, such as the scarcity of organ donors for HT and the risk of complications, including infection and thrombosis with MCS devices, creating a challenging issue for cardiologists, who must carefully evaluate each patient’s situation and decide whether and when to refer them for surgical intervention. It requires a high level of expertise and experience to make informed decisions and choose the most appropriate treatment option for each patient.

Several score-based models have been developed for heart failure patient referral, including the Heart Failure Survival Score (HFSS) [5] and Seattle Heart Failure Model (SHFM) [6]. Both models are multivariate proportional hazard survival models requiring not routinely collected data. In addition, these models are limited in their predictive ability for individual patients. Another class of popular methods that have been introduced to this field are based on machine learning and deep learning. These models have been widely used in general healthcare [7–9] and cardiovascular disease [10–14] and these have generated good model performance. However, deep learning models have the inherent issue of opacity and lack of justification for decision-making which has hindered their applications in medicine [15], where model interpretability allows clinicians to comprehend the rationale behind the model’s predictions, thereby facilitating the identification of new risk factors [16]. In addition, deep learning methods typically use a large number of parameters, which can be easily overfitted if the training sample is not large enough, another common challenge in medicine since large annotated training samples may not be available for rare diseases. Furthermore, the aforementioned machine learning methods only utilize numerical values, ignoring the rich information available through medical codes. The medical codes encode information regarding diagnosis, medications, and comorbidities, which are also informative for decision-making. Given the limitations of the aforementioned methods, there is a need for interpretable models that can effectively use the information contained in medical codes to predict patients warranting timeline referral to a heart failure and transplant cardiologist.

To overcome these limitations of existing methods, we propose two logistic regression (LR)-based models that could leverage the inherent structural information within the data to predict potential candidates for advanced therapies. The choice of LR as the base model is due to its simple structure, natural interpretability and predictive power [17–19]. In order to apply LR to medical code data, we employ word embedding techniques from natural language processing (NLP) to represent the individual medical codes as numerical vectors, which could be stacked into representation matrices and used as input features for LR. Instead of standard LR, we use logistic tensor regression (LTR) to utilize the underlying multilinear structural information, which could improve both model performance and flexibility [20]. Additionally, we adapted the positional encoding (PE) technique commonly used in NLP, such as in the transformer model [21], to address the irregular temporal information inherent in the dataset. Originally developed to model the relative position of words in sentences, the PE technique has also been utilized in several studies to capture the irregular time intervals between adjacent measurements [22–24]. Both of our proposed LTR models can produce weights for medical codes which measure their relative importance. In the first LTR model, the weights are defined globally, whereas in the second LTR model, the weights can vary at the patient level. Moreover, similar to LR, the proposed models are interpretable and allow us to evaluate feature importance using the weights. We were also able to confirm that the most important medical codes selected by the models are consistent with clinical expertise.

Overall, we propose two novel interpretable models that can integrate both irregular temporal and structural information to effectively predict patients warranting evaluation for heart failure advanced therapies, providing a more transparent, comprehensive, and accurate approach.

## Methods

### Overview of the proposed framework

**Fig. 1:**
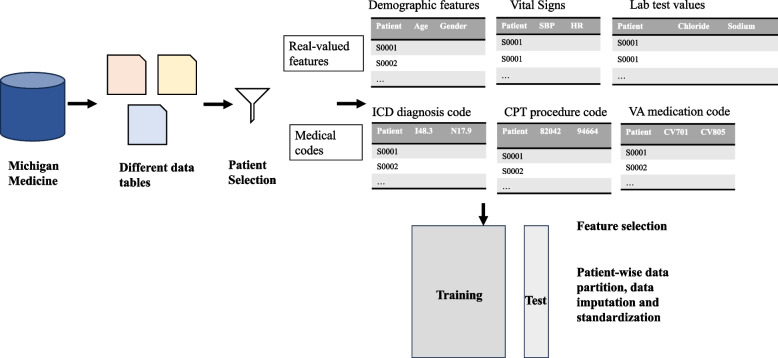
The workflow of our analysis: Medical codes such as ICD-10 diagnosis codes (e.g., I50.23), VA drug codes (e.g., CV701), and procedure codes (e.g., 80076) were collected along with lab test values to predict whether the patients were potentially in need of heart failure advanced therapies

**Fig. 2:**
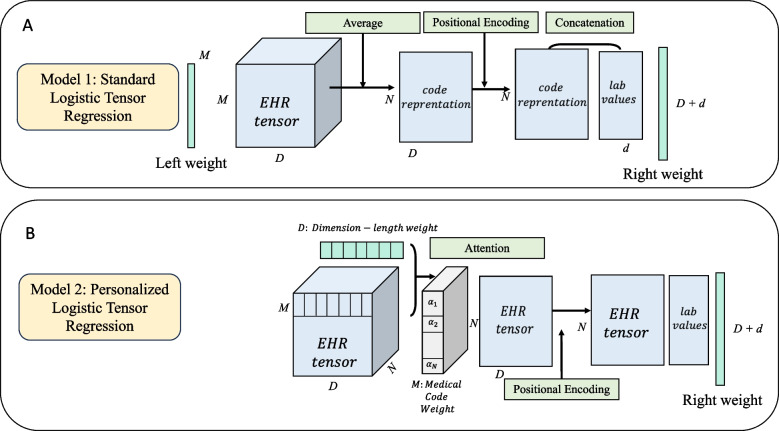
The proposed two interpretable algorithms. **A** The left weight in standard logistic regression learns the global weights for every medical code; **B** The dimension-length weight enables the algorithm to learn the weight for medical codes at individual level

In this study, we propose two LTR models, namely the Standard LTR model and the Personalized LTR model, to make predictions of the likelihood of HF patients requiring advanced therapies at the beginning of a visit based on features from their previous visit and the time between visits. Electronic health records (EHR) data comprising medical codes and lab test values were collected, and the medical codes were represented in an embedding space using the word2vec word embedding technique from NLP, which were then aggregated into an embedding matrix for each clinical visit. The input for both models is the same, which for the *i*th sample in the dataset consists of (1) the code embedding matrix: $\textbf{X}_i \in \mathbb {R}^{ M \times D}$ where *M* is the number of medical codes and *D* is the dimension of code embedding space, (2) selected important lab values: $\textbf{x}_i \in \mathbb {R}^{d}$ where *d* is the number of selected lab values, and (3) time elapsed measured in days: $t_{i}\in \mathbb {R}$ between the previous visit when the EHR data were collected and the next visit when the referral decision was made. The label is $y_i \in \{-1,1\}$ where 1 means the patient warrants evaluation for advanced therapies while -1 means the opposite. In the Standard LTR model, code weights can be learned directly from the model and evaluated globally, while in the Personalized LTR model, code weights are learned based on the attention mechanism and can be interpreted individually. Subsequently, a global context vector can be computed based on the code weights, and all medical code representations undergo weighted aggregation to form the final visit representation in the form of an embedding matrix. During model training, we also append the lab values to the visit representation and inject the irregular temporal information is using PE. The overall framework is illustrated in Fig. 1 and the schematic illustration of the two proposed algorithms are depicted in Fig. 2.

### Dataset

This study utilized a dataset obtained from Michigan Medicine, which was approved by the University of Michigan’s Institutional Review Board (IRB) under protocol number HUM00184418 and the need for informed consent was waived. The inclusion criteria for the end-stage heart failure patients included:

- At least two hospitalization admissions for heart failure between January 1, 2013 and June 30, 2021
- Adult patients who were $\ge$ 18 years and $\le$ 80 years of age at the time of admission
- Most recent ejection fraction was $\le$ 35% by echocardiography
- Body mass index (BMI) $\le$ 50 $\textrm{kg}/\textrm{m}^2$

Each training sample consisted of a pair of consecutive visits. In order to expand the dataset, *n* consecutive visits of the same patient were treated as $n-1$ separate pairs of consecutive visits. This approach yielded a total of 300 patients and 557 paired visit samples. The label $y_i \in \{0,1\}$ for each paired visit was determined by the care they received at the time of the second visit. The data were then grouped into two categories: (1) patients who received advanced therapies at the time of their second visit, i.e. $y_i = 1$ and (2) patients too well for advanced therapies, defined as those who lived at least two years after a heart failure hospitalization without receiving advanced therapies, i.e. $y_i = 0$. To prevent data leakage, we employed patient-wise data splitting to train and validate the model.

### Medical code embedding

Medical codes contain crucial information about a patient’s medical history that can aid in predicting disease progression. However, these codes cannot be directly analyzed due to their string format. To overcome this issue, we utilized word2vec [25, 26], a popular unsupervised word embedding algorithm in NLP, to map each medical code to a vector in a user-defined vector space. Word2vec is based on shallow neural network models that learn word embeddings by either predicting a target word from its context, i.e. continuous bag of words (CBOW), or predicting context words from a target word, i.e. skip-gram with negative sampling (SGNS). Each code is considered as a medical word, and a hospital visit with multiple codes is treated as a medical document.

To ensure that no individual medical code was highly correlated with whether the patient received advanced therapies, we calculated the Pearson correlation coefficient between each medical code which is represented as a binary variable and the label $y_i$. The highest correlation coefficient was 0.51, corresponding to the procedure for electrocardiogram. As a result, all codes were kept for downstream analysis.

To validate the quality of the medical code embeddings, Phecode, which was originally developed for phenome-wide association studies, is adapted for quantitative evaluation [27]. Phecode can group similar ICD codes into meaningful clinical phenotypes. For example, the ICD codes: *I50.20* and *I50.21* share the same Phecode *428.3*. Utilizing this mapping relationship, we defined a binary classification problem for pairs of medical codes based on if the two codes share the same Phecode, and predicted the class for each pair or medical codes using the cosine similarity of their vector representations. Under our circumstances, the positive pairs make up less than 1% in all pairs, making specificity and sensitivity non-informative. By tuning the threshold for categorizing the cosine similarity, specificity and sensitivity change drastically; therefore, AUC is used to evaluate medical code embeddings.

In addition to the main features extracted from medical codes, we also selected a set of *d* lab features recommended by cardiologists and which are summarized in Table 1. The features for each training sample could be combined into a code embedding matrix $\textbf{X}_i \in \mathbb {R}^{ M \times D}$ where *M* is the total number of medical codes and *D* is the embedding dimension by stacking the code embedding vectors along the rows and multiplying by row-wise one-hot encoding, and a lab value vector $\textbf{x}_i \in \mathbb {R}^{d}$.

**Table 1:** Clinical characteristics of heart failure patients included in the model

	Features	Units	Positive	Negative
Demographics	Ages	years	53(14)	58 (14)
	Male Gender	%	81.3	70.3
Vital signs	Systolic blood pressure	mmHg	$102.74 \pm 14.95$	$121.68 \pm 22.74$
	Heart rate	bpm	$86.09\pm 18.85$	$86.15\pm 16.68$
Lab metabolites	Chloride	mmol/L	$100.62\pm 4.88$	$101.60\pm 5.79$
	Sodium	mmol/L	$137.48\pm 3.58$	$138.06\pm 4.35$
Cormorbidity	Diabetes	%	43.00	62.10
	Hypertension	%	67.40	89.30

Clinical characteristics of patients requiring HT/LVAD evaluation (“Positive”) and those too well for HF advanced therapies (“Negative”). Displayed are the mean (standard deviation) for continuous variables and N (%) for comorbidities

### Temporal information encoding

In EHR data analysis, temporal information is critical to modeling patient health trajectories accurately. The most common way to incorporate temporal information in EHR data is to use Recurrent Neural Networks (RNN) or some variant, such as the Long Short Term Memory (LSTM) model [28–30]. However, these models strongly assume that the time intervals between adjacent visits are consistent or regularly sampled, which does not apply to our research question. In our study, we encountered the issue where the patients had irregularly spaced visits, making it unsuitable to apply these methods to incorporate temporal information. Instead, we adapted the PE technique commonly used in NLP to deal with temporal irregularity within the sampled measurements [21]. The PE technique encodes the relative position of words within a sentence in the form of the angle of a rotation matrix applied to the embedding space. In this study, we adapted the PE technique by encoding the elapsed time $t_i$ between two consecutive visits of the *i*th sample along the time domain with sine and cosine functions. The formula for the *PE* function is detailed below:

$$\begin{aligned} PE(t_i, 2j-1){} & {} = \sin \left( \frac{t_i}{10000^{2j/(D+d)}}\right) \\ PE(t_i, 2j){} & {} = \cos \left( \frac{t_i}{10000^{2j/(D+d)}}\right) \end{aligned}$$

where *j* ranges from 1 to $(D+d)/2$ and the embedding dimension *D* is chosen such that $D+d$ is even, and we multiplied the *D* columns of the embedding matrix $\textbf{X}_i$ and the *d* entries of the lab vector by the *PE* function to encode a meaningful and robust representation of time for downstream analysis.

### Standard logistic tensor regression

In this section, we describe the Standard LTR model in our study. The sample is represented as $\{\textbf{X}_{i}, \textbf{x}_i, y_{i}\}$ for $i = 1,\dots , N$, where $\textbf{X}_{i} \in \mathbb {R}^{M \times D}$, $\textbf{x}_i\in \mathbb {R}^d$, *N* is the number of samples, and $y_{i} \in \{-1,1\}$ is the corresponding label.

Generalizing the standard LR classifier

$$\begin{aligned} f(\textbf{x})=\frac{1}{1+\exp \left( -\textbf{x}^\top \textbf{v}-b\right) } \end{aligned}$$

for a vector-valued testing sample $\textbf{x}$ where $\textbf{v}$ denotes the vector of coefficients and *b* denotes the scalar intercept of the model, the LTR classifier adapted to our training samples could be represented as:

$$\begin{aligned} f_{\textrm{tensor}}(\textbf{X},\textbf{x})=\frac{1}{1 + \exp \left( -\left[ \textbf{u}^\top \textbf{X}~|~\textbf{x}^\top \right] \textbf{v} - b\right) } \end{aligned}$$

where $\textbf{u} \in \mathbb {R}^{M}$ is the weights for the *M* medical codes, $\textbf{v} \in \mathbb {R}^{D+d}$ is the vector of coefficients in the regression model and $b \in \mathbb {R}$ is the intercept. The LTR classifier is an extension of the standard LR classifier to higher-dimensional feature arrays.

The parameters $\textbf{u}$, $\textbf{v}$ and *b* can be estimated by solving the optimization problem

$$\begin{aligned} \left( \widehat{\textbf{u}}, \widehat{\textbf{v}},\widehat{b}\right) =\arg \min _{\textbf{u},\textbf{v}, b}\mathcal {L}(\textbf{u},\textbf{v}, b) \end{aligned}$$

where the loss function

$$\begin{aligned} \mathcal {L}(\textbf{u},\textbf{v}, b)=-\sum \limits _{i=1}^{N} \log \left( 1+\exp \left( -y_{i}\left( \left[ \textbf{u}^\top \textbf{X}_i~\big |~\textbf{x}_i^\top \right] \textbf{v} + b\right) \right) \right) \end{aligned}$$

is the negative log-likelihood function.

### Personalized logistic tensor regression

The limitation of the LTR model formulated above is that the interpretation of medical codes is at the population level. In order to evaluate the importance of medical codes at the sample level, in this section we propose a Personalized LTR model based on sample-wise inner-product matrices of size $D\times D$ instead.

To this end, define the *i*th sample-wise inner-product matrix $\textbf{S}_i\in \mathbb {R}^{D\times D}$ by the formula

$$\begin{aligned} \textbf{S}_i=\textbf{X}_i^\top \textbf{X}_i, \end{aligned}$$

and consider the samples represented as $\{\textbf{S}_i, \textbf{x}_i, y_i\}$ instead of $\{\textbf{X}_i, \textbf{x}_i, y_i\}$ for $i = 1,\dots ,N$. Working with the matrices $\textbf{S}_i$ rather than the embedding matrices $\textbf{X}_i$ allows us to drastically reduce the model dimensionality from $M\times D$ to $D\times D$ without losing too much information. The reduction in dimensionality is possible since the matrices $\textbf{X}_i$ only contain non-zero rows corresponding to the medical codes from the *i*th paired visit sample, which could be recovered from the matrices $\textbf{S}_i$ if the number of non-zero rows (i.e. medical codes) is much smaller than the embedding dimension *D*. The more naive approach of simply deleting the zero rows from the matrix $\textbf{X}_i$ does not work since the number of rows is different across the training sample.

Applying the same LTR model to the sample matrices $\textbf{S}_i$ instead of $\textbf{X}_i$ leads to a different set of parameters $\textbf{w}\in \mathbb {R}^D$, $\textbf{v}\in \mathbb {R}^{D+d}$ and $b\in \mathbb {R}$ with loss function

$$\begin{aligned} \mathcal {L}(\textbf{w},\textbf{v}, b)=-\sum \limits _{i=1}^{N} \log \left( 1+\exp \left( -y_{i}\left( \left[ \textbf{w}^\top \textbf{S}_i~\big |~\textbf{x}_i^\top \right] \textbf{v} + b\right) \right) \right) \end{aligned}$$

where $\textbf{w}$ is the *D*-dimensional vector consisting of the weights for the word embedding space $\mathbb {R}^D$. Another way to understand the new weight vector $\textbf{w}$ is to note that the loss function could be equivalently written as

$$\begin{aligned} \mathcal {L}(\textbf{w},\textbf{v}, b){} & {} =-\sum \limits _{i=1}^{N} \log \left( 1+\exp \left( -y_{i}\left( \left[ \textbf{w}^\top \textbf{X}_i^\top \textbf{X}_i~\big |~\textbf{x}_i^\top \right] \textbf{v} + b\right) \right) \right) \\{} & {} =-\sum \limits _{i=1}^{N} \log \left( 1+\exp \left( -y_{i}\left( \left[ \textbf{u}_i^\top \textbf{X}_i~\big |~\textbf{x}_i^\top \right] \textbf{v} + b\right) \right) \right) \end{aligned}$$

where

$$\begin{aligned} \textbf{u}_i=\textbf{X}_i\textbf{w}\in \mathbb {R}^M \end{aligned}$$

is the weight for the *M* medical codes as in the previous model. Therefore the new weight vector $\textbf{w}$ leads to weight vectors $\textbf{u}_i$ in the Standard LTR model which are allowed to vary on an individual level while depending on a much smaller number of parameters.

From another perspective, the proposed algorithm can be explained and computationally implemented with an attention mechanism. The attention mechanism is now widely used in EHR data analysis [24, 31, 32]. It allows the network to prioritize the information on specific inputs by assigning different weights, which not only helps to improve the model accuracy but also facilitates the interpretation of complex inputs. In the framework of the global attention mechanism [33], our proposed algorithm could be formulated as follows:

To learn different weights for medical codes, the variable-length vector $\textbf{u}$ and a $\tanh$ function is applied. The formula to calculate weight is illustrated below:

$$\begin{aligned} \left[ u_1,u_2,...u_M\right] =\textbf{X}_i\textbf{w}. \end{aligned}$$

Given the patient matrix $\textbf{X}_i$ and the learned weights $\alpha _i$, a patient-wise representation $\mathbf {c_i}$ could be constructed as

$$\begin{aligned} \mathbf {c_i}=\left[ u_1,u_2,...u_M\right] \textbf{X}_i. \end{aligned}$$

Provided with a patient-wise vector, predicted probability can be computed as

$$\begin{aligned} \hat{y_i}=\tanh \left( \left[ \mathbf {c_i}\big | \textbf{x}_i^{\top }\right] \textbf{v}+ b\right) . \end{aligned}$$

### Model solving and model training

Gradient descent optimization algorithms were used to solve both models. The algorithm for Personalized LTR can be found in Algorithm 1, from which the algorithm for Standard LTR can also be easily adapted by replacing $\textbf{w}_{i}$ by $\textbf{u}_{i}$ and removing line 2.

**Figure:**
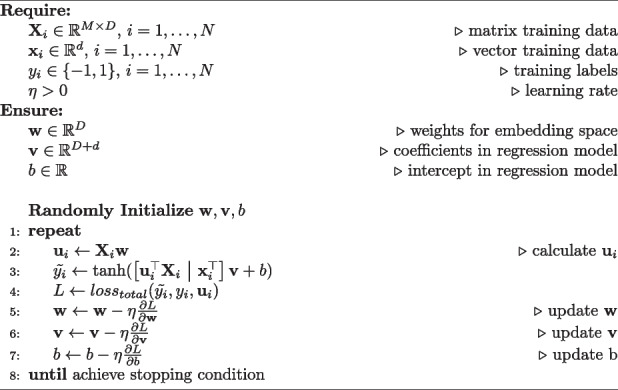
**Algorithm 1** Personalized logistic tensor regression – solving with gradient descent optimization

Besides the regular cross-entropy loss function, an *l*1 norm-based regularization on the weights for medical codes is also added to induce sparsity. The loss function is written as:

$$\begin{aligned} loss_{total} = loss_{ce} + \lambda \Vert \textbf{u}\Vert _{1} \end{aligned}$$

where $\lambda$ controls the regularization effect. This penalization encourages the model to focus on a small number of important codes. $loss_{ce}$ is calculated as below:

$$\begin{aligned} loss_{ce}=-\frac{1}{N} \sum \limits _{n=1}^N y_i \log \left( \hat{y}_i\right) +\left( 1-y_i\right) \log \left( 1-\hat{y}_i\right) \end{aligned}$$

where N is the total number of samples, $y_i$ is a binary response and $\hat{y}_i$ is a real-valued number ranging from zero to one representing the probabilities of the sample assigned to each class.

The two proposed algorithms are trained by back-propagation with an adaptive moment estimation (Adam) optimizer. All the trainable parameters in our proposed algorithms were initialized with Kaiming uniform distributions and the optimal hyperparameter combinations were generated using an exhaustive grid search.

### Baseline models and inputs

During our analysis, we determined that M equals 8438, while D was selected as 100, and d was set to 4. For comparison, we built models as follows:

- Logistic Regression, support vector machine (SVM) with linear kernel, gaussian Naive Bayes (NB) and random forest (RF) were chosen as the baseline for comparison. For the training tensor of size $N \times M \times D$, a global pooling is performed along the medical codes axis, taking the average over the *M* medical codes. In this way, every sample is represented by a *D*-dimensional vector. Besides, lab values were also considered and concatenated. Hence, the input is a 104-dimensional vector representing a patient’s medical history and lab test results.
- Standard Logistic Tensor Regression: The input is an $N \times M \times D$ tensor and a $N \times d$-dimensional lab matrix. In addition, positional encoding is utilized to incorporate irregular temporal information into the tensor.
- Personalized Logistic Tensor Regression: The input is the same as the one for Standard LTR.

### Evaluation metrics

Accuracy, F1 score, Area under the receiver operating characteristic Curve (AUC) and Area Under the Precision-Recall Curve (AUPRC) were computed for model evaluation. Accuracy and F1 score are defined as:

$$\begin{aligned} \textrm{Accuracy}{} & {} = \frac{\textrm{T P}+\textrm{T N}}{\textrm{T P}+\textrm{T N}+\textrm{F P}+\textrm{F N}} \\ \textrm{F1}{} & {} = \frac{2 \times \textrm{TP}}{\textrm{FN} +2 \times \textrm{TP} + \textrm{FP}} \end{aligned}$$

where TP = true positive, TN = true negative, FP = false positive and FN = false negative.

## Experiments and results

### Embedding qualities

**Fig. 3:**
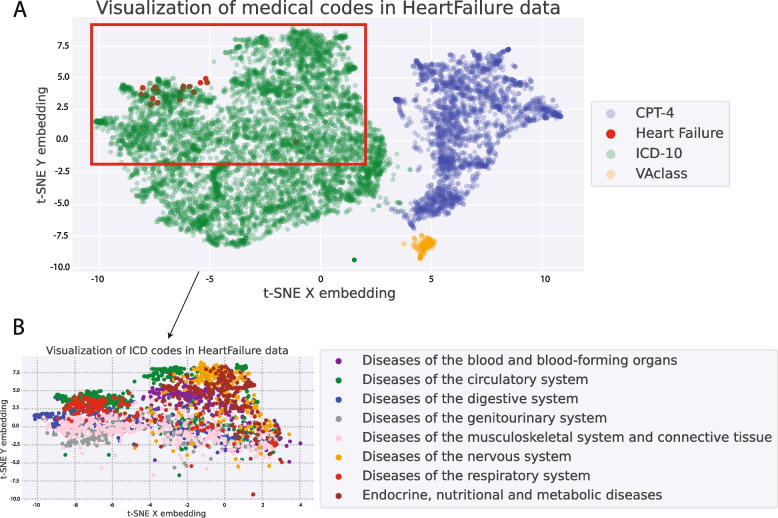
Embeddings for medical codes: Fig. 3A visualizes the embeddings of medical codes of three main categories (CPT, VA, ICD). Figure 3B visualizes the embeddings of ICD codes extracted from the red rectangular box from Fig. 3A

Word2vec was applied to 8438 unique medical codes, including 5970 diagnosis codes (ICD-10), 2229 procedure codes (CPT-4), and 239 drug codes (VA Drug Class), to generate code embeddings. Figure 3A displays all the medical codes, while Fig. 3B depicts only subsets of ICD diagnosis codes colored by the system. Notably, the codes from the same category or system were observed to cluster together, and different clusters were clearly separated in both figures. The co-localization of diagnosis codes in the figure qualitatively validated our assumption that the word representation learned from the word2vec algorithm can capture meaningful latent information.

The results of our embedding performance are listed in Table 2. Based on our analysis, we determined that the embeddings obtained from SGNS, with a dimension of 100, were the most suitable inputs for our model.

**Table 2:** Embedding performance (AUC) varies with dimensions and algorithms

Methods	dim = 80	dim = 100	dim = 150	dim = 200
**SGNS**	0.72	0.72	0.72	0.71
**CBOW**	0.65	0.65	0.65	0.65

### Heart failure patients prediction

In order to evaluate the performance of the proposed model, we utilized a patient-wise stratified ten-fold cross-validation technique. The entire dataset was first partitioned into training and test datasets, with a test ratio of 0.2. The training dataset was further divided into ten folds of approximately equal size. During each iteration of the ten-fold cross-validation process, one fold was designated as the test dataset, while the other nine folds served as the training dataset. This process was repeated ten times, and the model’s performance and robustness were evaluated by calculating the average and standard deviation. We then compared the predictive performance of the proposed model with that of the baseline approaches.

The comparative results are shown in Table 3. From Table 3, Standard LTR achieves an F1 score of 0.708, AUC of 0.903 and AUPRC of 0.836, while Personalized LTR had an F1 score of 0.670, AUC of 0.869 and AUPRC at 0.839. These two models showed much higher F1 scores, AUC and AUPRC compared to all the other traditional machine learning methods which do not take structural information into account. In terms of other metrics, the models also showed superior results. Additionally, Standard LTR showed strong model robustness concerning the F1 score and AUC. In particular, when compared against LR, two LTR-based models improved the performance by a large margin, demonstrating the benefits of taking structural information into account.

We also performed a comparison of the effectiveness of various machine learning methods against Standard LTR and Personalized LTR, using Cohen’s D as a metric [34]. Cohen’s D is a standardized effect size measure that is commonly used in statistical analysis to express the magnitude of a difference or effect between two groups. Empirically, a Cohen’s D value above 0.8 is interpreted as indicating a large effect, while values between 0.5 and 0.8 suggest a moderate effect. This comparison is detailed in Table 3. It was observed that Standard LTR significantly outperformed other models, showing a large effect, while Personalized LTR demonstrated a moderate to large effect.

**Fig. 4:**
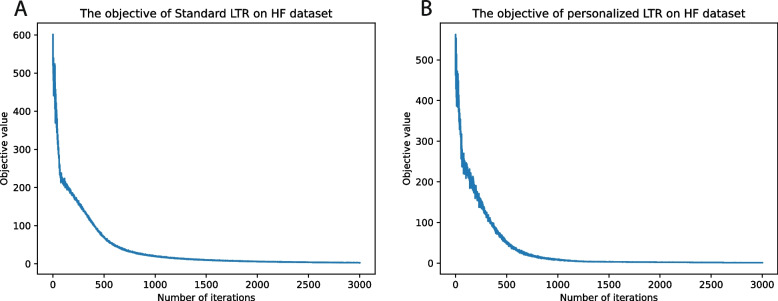
The objective value of two proposed models: The two proposed models converges after around 2000 steps

Besides, both models converged and Personalized LTR converged in fewer iterations than Standard LTR, as illustrated in Fig. 4.

**Table 3:** Comparison of 10-fold Cross-validation Model Performance (mean±std)

	Accuracy	F1	AUC	AUPRC	Cohen’s D ($S\vert P$)
**S-LTR**	0.832 (0.044)	**0.708 (0.086)**	**0.903 (0.047)**	0.836 (0.11)	
**P-LTR**	0.826 (0.055)	0.670 (0.12)	0.869 (0.087)	**0.839 (0.11)**	
**LR**	0.828 (0.049)	0.642 (0.15)	0.841 (0.11)	0.811 (0.13)	$0.733\ \vert \ 0.282$
**SVM**	0.837 (0.052)	0.657 (0.15)	0.823 (0.091)	0.792 (0.13)	$1.104\ \vert \ 0.517$
**Gaussian NB**	0.744 (0.066)	0.603 (0.13)	0.795 (0.10)	0.747 (0.15)	$1.382\ \vert \ 0.790$
**RF**	**0.844 (0.041)**	0.656 (0.14)	0.829 (0.096)	0.803 (0.13)	$0.979\ \vert \ 0.437$

* S-LTR Standard LTR, *P-LTR* Personalized LTR, Cohen’s D ($S\vert P$): Cohen’s D is computed by comparing the AUC values of various models with those of S-LTR and P-LTR model

### Weight transferring from LTR models

In addition to achieving better results in themselves, the weights obtained through Standard and Personalized LTR for visit representation can be transferred to other machine-learning models. To examine the effectiveness of the weight transfer, we collected the ten sets of weights for the medical code in our previous ten-fold cross-validation experiments and then utilized them to aggregate the medical codes. Mean and standard deviations were calculated to compare with the original model, which was trained without weight transfer. As shown in Table 4, all model performances were improved compared to the original model. The improvement is significant, as evidenced by the large effect sizes calculated using Cohen’s D. Specifically, the F1 score, AUC, and AUCPR for SVM models increased by 4.57%, 8.87%, and 8.33%, respectively, while the F1 score, AUC and AUPRC for RF increased by 4.57%, 7.24%, and 5.85%, respectively. Although the AUC decreased by 12.58% for Gaussian NB, the F1 score and AUPRC increased by 15.26% and 11.38%, respectively. Notably, using the learned weights for training reduced the standard deviation, indicating improved model stability.

**Table 4:** Comparison of model performances incorporating weight transfer(mean±std)

	Methods	Accuracy	F1	AUC	AUPRC	Cohen’s D
**SVM**	standard	0.837 (0.052)	0.657 (0.15)	0.823 (0.091)	0.792 (0.13)	
	S-weighted	0.839 (0.038)	0.687 (0.042)	**0.896 (0.021)**	0.858 (0.052)	1.05
	P-weighted	**0.853 (0.051)**	**0.698 (0.10)**	0.882 (0.041)	**0.874 (0.046)**	0.836
**Gaussian NB**	standard	0.744 (0.066)	0.603 (0.13)	0.795 (0.10)	0.747 (0.15)	
	S-weighted	**0.839 (0.034)**	0.695 (0.046)	0.695 (0.046)	**0.832 (0.083)**	1.274
	P-weighted	0.829 (0.025)	**0.715 (0.043)**	**0.871 (0.037)**	0.794 (0.077)	1.008
**RF**	standard	0.844 (0.041)	0.656 (0.14)	0.829 (0.096)	0.803 (0.13)	
	S-weighted	0.839 (0.034)	0.686 (0.046)	**0.889 (0.032)**	0.850 (0.054)	0.839
	P-weighted	**0.852 (0.051)**	**0.701 (0.10)**	0.888 (0.030)	**0.867 (0.042)**	0.830

*S-weighted The weights learned from Standard LTR were used to generate visit representation, *P-weighted* The weights learned from Personalized LTR were used to generate visit representation; Cohen’s D is calculated by comparing the AUC values of the standard model to its weight transferred models

### Model interpretation

Besides achieving superior model performance, LTR can also facilitate the interpretation of the medical codes in the dataset. The Standard LTR model can provide information on the population-level importance of the codes, while the Personalized LTR model can capture individual-level weights. To evaluate the weights extracted from the two models, we retrained them using the entire training dataset and visualized the weights obtained from the models.

The weights obtained from both models range from -1 to 1 with signs indicating their positive or negative association with the outcome. A positive sign suggests that the presence of the medical code is associated with receiving advanced therapies, while a negative sign indicates the opposite. Regarding population-level interpretation, our study specifically focused on the diagnosis codes. Out of a total of 8438 codes, 2336 had a positive effect greater than 0.1. To avoid the influence of coincidental cases in our relatively small dataset, we only considered codes that were associated with at least thirty patients. This reduced the number of codes to 17, and the top 5 most essential codes are presented in Table 5. According to Table 5, the most predictive codes of the need for heart failure advanced therapies at a subsequent visit included chronic kidney disease, hypotension, pulmonary heart disease, and mitral regurgitation, and atherosclerotic heart disease. Chronic kidney disease is one of the most common comorbidities in end-stage heart failure patients [35, 36]. In addition, hypotension and mitral regurgitation are vital clinical clues of advanced heart failure [35]. The presence of pulmonary hypertension has been linked to poor clinical outcomes in patients with end-stage heart failure [37]. Furthermore, atherosclerotic heart disease has a known association with heart failure-related death [38]. The top codes and their rankings are consistent with the expertise of clinicians and the literature review.

**Table 5:** Top 5 diagnosis groups with high weights in HF patients

Index	Diagnosis groups	Weights
1	Chronic kidney disease	0.954
2	Hypotension	0.943
3	Pulmonary heart disease	0.607
4	Mitral regurgitation	0.312
5	Atherosclerotic heart disease	0.281

**Fig. 5:**
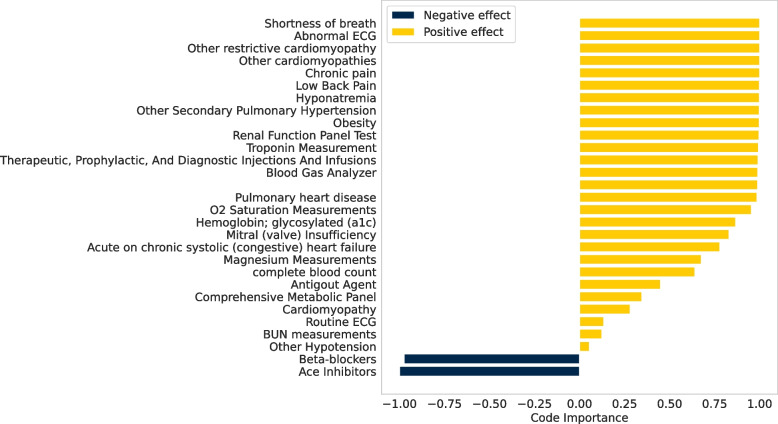
Example Personal Interpretation of Medical codes: The X-axis extends from -1 to 1, indicating the extent and direction of influence on the result. Positive contributions are signified by yellow, while negative influences are denoted by blue. The Y-axis lists medical codes in order of significance

To obtain a more nuanced understanding of how medical decisions differ among patients in a population, we utilized the Personalized LTR model to evaluate the significance of medical codes at the individual patient level. This approach is similar to the Standard LTR model and enables us to measure how much each medical code positively or negatively contributes to the prediction outcome. To demonstrate the effectiveness of this approach, we selected one patient from the testing dataset and presented a corresponding bar plot in Fig. 5, which shows the importance of the medical codes. The weights in the accompanying figure indicate the relative importance of different medical codes, with their signs indicating the direction and magnitude of their impact. In this specific case, the patient had been diagnosed with cardiomyopathy, hyponatremia, and pulmonary heart disease and was experiencing symptoms of chronic pain and shortness of breath. The comorbidities and symptoms listed above are associated with end-stage heart failure [35, 39, 40]. Additionally, the patient had undergone various medical procedures, including oxygen saturation measurements and renal function tests. Although they are screening tests for admitted patients, it indicates the results come from these procedures need more attention [41, 42]. Our analysis also identified intolerance to beta-blockers of angiotensin-converting enzyme (ACE) inhibitors as a marker of advanced heart failure, which has known associations with advanced heart failure in the published literature [35]. Overall, these diagnoses and procedures were highly positively correlated with advanced heart failure management, whereas the medications had the opposite effect, providing valuable insights into the patient’s condition.

**Fig. 6:**
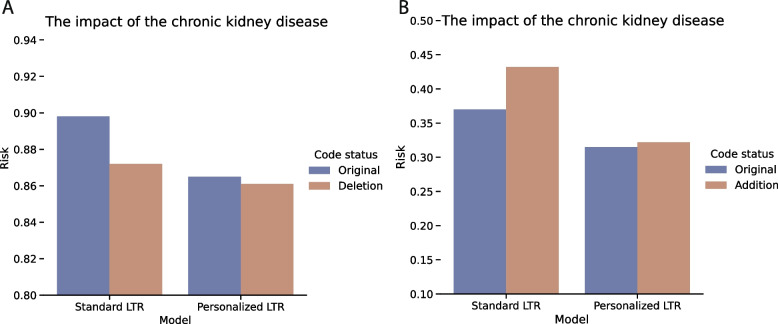
Simulations on patients with weights learned from Standard LTR: (1) We randomly selected a patient in cohort 2 and added the medical code N18.2 for chronic kidney disease; (2) The randomly selected patient in cohort 1 deleted N18.2

For another illustration of the interpretability property of the weights associated with the medical codes, we undertook two simulations to identify the quantitative contributions of the chronic kidney disease code toward the risk of receiving advanced therapies. Specifically, we investigated the impact of (1) adding the risk code to patients who did not possess it originally and (2) deleting the risk code from patients who already had it. Figure 6 illustrates the outcomes of these simulations. We observed that the Standard LTR model’s predicted risk decreased from 0.898 to 0.865, while the Personalized LTR model’s predicted risk decreased from 0.865 to 0.861 when the risk code was removed from patients deemed urgent for advanced heart failure therapies. In contrast, when the risk code was added to patients considered too well for advanced therapies, the Standard LTR model’s predicted risk increased from 0.370 to 0.432, while the Personalized LTR model’s predicted risk increased from 0.315 to 0.322. The observed behavior of both models indicates a positive correlation between chronic kidney disease and worsening heart failure, thereby validating the efficacy of our proposed LTR models.

## Discussion

### General

In this study, we proposed two LTR models that aim to predict the potential eligibility of advanced therapies for heart failure patients and to evaluate the importance of medical events at both the population and individual levels. These models were trained and validated using data collected from an academic medical center. To benchmark our models, we compared them with other machine learning methods. Our Standard LTR algorithm reported an F1 score of 0.667, an AUC of 0.904 and an AUPRC of 0.873, while the proposed Personalized LTR achieved an F1 score of 0.670, an AUC of 0.869 and an AUPRC of 0.839, outperforming all the baseline methods. Moreover, the weights learned from our two LTR models can be transferred to other machine-learning models, improving the performance of these models. Additionally, the magnitude of the weights can be interpreted as their relative importance while the sign imposes directionality on the weights. To our knowledge, this is the first model that employs the medical codes for HF advanced therapies eligibility prediction.

Taking into account structural information not only enhances the model’s performance but also increases its robustness. Both LTR models consider the importance of medical codes and employ code weights to construct the visit representation. Compared to standard vector-based LR, which averages all medical code embeddings as the visit representation, our proposed tensor-based models effectively utilize the multilinear structure to achieve performance significantly surpassing that of ensemble methods and other traditional machine-learning techniques. Although the weights are learned under the LTR framework, they can also be transferred to other models to enhance performance. Whether the weights come from Standard LTR or Personalized LTR, they facilitate model training and significantly improve performance, supporting our claim that modeling the internal structural information of medical codes is beneficial [20, 43, 44]. Machine learning models with weights transferred from Personalized LTR generally performed better than those with weights from Standard LTR, possibly because Personalized LTR evaluates medical events individually, allowing for more flexibility.

Our two models complement each other regarding interpretability. Standard LTR provides insights into the population-level importance of medical codes. It can identify associated symptoms and diseases that may impact the heart failure population, serving as an inspiration for finding potential risk factors. This can help cardiologists recognize their illness severity and potentially initiate an evaluation for advanced HF therapies as appropriate. From a patient-level perspective, personalized interpretation creates an individualized heart failure profile, providing basis for finding patient-specific risk factors. Overall, our proposed models exhibit superior performance and enhanced interpretability compared to traditional machine learning methods, thus representing a promising avenue for identifying patients in need of advanced HF therapies.

### Limitations

It is important to acknowledge that our study has several limitations. First, our dataset was relatively small compared to the vast number of medical codes, resulting in high standard deviations across models. Despite the fact that our model has a simpler structure while maintaining structural information, the standard deviations remain relatively high. In addition, our models heavily relied on the quality of the medical codes, which may be inaccurately recorded or undetected. Errors in medical codes can impact results as differences between patients who are too well for HT/MCS and those who require urgent treatment are subtle. Therefore, it would be important in the future to investigate how to incorporate more clinical measurements in conjunction with medical codes to enhance model performance and validity. Furthermore, the data was collected from only one institution, which may decrease the generalizability of our findings and future studies are needed using data from multiple institutions.

Apart from the potential issues related to the data, our models still have the potential to improve by accounting for further structural information within the data. Currently, we only utilized two visits for modeling: features were extracted from a single hospitalization and used to predict the need for advanced therapies in a subsequent hospitalization. Since heart failure is a chronic disease, incorporating additional longitudinal data would be clinically advantageous. Furthermore, the relationships among codes of different categories are also important but have not been studied in our work. There have been several works using graphical models to account for the interaction among different medical codes [44–46]. Therefore, other more powerful methods incorporating these additional structures could be applied to the advanced heart failure population.

## Conclusion

Our study proposed two LTR models: Standard and Personalized versions for predicting potential eligibility for advanced therapies for HF patients based on the previous clinical features and irregular temporal information. These models incorporated both structural and temporal information present in EHR medical codes while maintaining a simple learning structure to assess the importance of clinical events both globally and individually. Our results demonstrated that our methods outperformed existing models, indicating that the inclusion of structural information can improve predictive performance and provide additional useful insights to enhance interpretability. Furthermore, the weight importance learned by our models aligns well with clinical practice and literature, highlighting their potential value for future research in the field of heart failure. In the future, we will further explore the application of this model in other healthcare areas, not limited to heart failure. In addition, since determining who should be referred to advanced therapies is challenging, physicians’ confidence in their labels should possibly be incorporated into the model as privileged information for better clinical diagnosis.

## Data Availability

The UM data in this article cannot be shared publicly because it contains privacy and protected health information of the subjects. Researchers who are interested in getting Michigan Medicine data should contact https://PHDataHelp@umich.edu for guidance. The codes for the two logistic tensor regression models are publicly available: https://github.com/kayvanlabs/Generalized-logistic-tensor-regression.

## Abbreviations

AUC: Area under the curve
ACE: Angiotensin-converting enzyme
AUPRC: Area under the precision-recall curve
BMI: Body mass index
CBOW: Continuous bag of word
EHR: Electronic health records
IRB: Institutional review board
LTR: Logistic tensor regression
LR: Logistic regression
LSTM: Long short-term memory
NB: Naive Bayes
NLP: Natural language processing
PE: Positional encoding
RF: Random forest
RNN: Recurrent neural networks
SGNS: Skip-gram with negative sampling
SVM: Support vector machine
